# RNF180 mediates STAT3 activity by regulating the expression of RhoC via the proteasomal pathway in gastric cancer cells

**DOI:** 10.1038/s41419-020-03096-3

**Published:** 2020-10-20

**Authors:** Zizhen Wu, Huifang Liu, Weilin Sun, Yingxin Du, Wenting He, Shiwei Guo, Liqiao Chen, Zhenzhen Zhao, Pengliang Wang, Han Liang, Jingyu Deng

**Affiliations:** 1 Department of Gastric Cancer, Tianjin Medical University Cancer Institute and Hospital, National Clinical Research Center for Cancer, Tianjin, China; 2 Key Laboratory of Cancer Prevention and Therapy, Tianjin, China; 3 Tianjin’s Clinical Research Center for Cancer, Tianjin, China

**Keywords:** Gastric cancer, Gastric cancer

## Abstract

Ring finger protein 180 (RNF180) is an important member of the E3 ubiquitin ligase family. As a tumor suppressor gene, RNF180 is significantly associated with the prognosis of patients with gastric cancer (GC) and can inhibit the proliferation, invasion, and migration of GC cells. Signal transducer and activator of transcription 3 (STAT3) are considered one of the most common oncogenes in human cancers with a key role in GC progression. In this study, we explored the molecular signaling pathways by which RNF180 could potentially regulate STAT3 through transcriptomics and proteomics experiments. Here, we found RNF180 overexpression could suppress STAT3 phosphorylation in GC cells. Ubiquitin label-free experiments showed that the ubiquitination level of Ras homolog gene family member C (RhoC) is significantly increased in GC cells transfected with an RNF180 expression vector (RNF180-GFP vector) compared with cells transfected with an empty vector (vehicle vector). We subsequently demonstrated that RNF180 could directly combine with RhoC and promote the ubiquitination and degradation of RhoC protein in GC cells. The phosphorylation level of STAT3 significantly decreased in GC cells after RhoC knockdown using small hairpin RNA (shRNA). Together, these results reveal RNF180 could inhibit GC progression by reducing the phosphorylation of STAT3 via the ubiquitination and degradation of RhoC protein in GC cells. Thus, the protein may be considered a novel therapeutic target for patients with GC.

## Introduction

Gastric cancer (GC) is one of the most common malignant tumor and the third leading cause of cancer-related mortality worldwide^1^. Diagnostic and therapeutic strategies for GC have seen remarkable improvements over the years. However, the 5-year survival rate of patients with the disease remains low due to the high incidence of metastasis. While radical surgery is considered the most effective treatment for GC, targeted therapy is gradually showing excellent therapeutic effects, especially for patients with unresectable GC. Therefore, the molecular mechanisms involved in the development and progression of GC should be explored.

Ring finger protein 180 (RNF180) is a novel discovered member of the ring finger protein family and has been confirmed to be ubiquitin ligase (E3), which is involved in many important physiological processes in vivo including cell growth, differentiation, and tumorigenesis. As a tumor suppressor gene, RNF180 also has a certain expression level in the cells of the normal body, and its expression also has important physiological functions. Under abnormal conditions, the body is affected by a variety of physical, chemical, and biological factors, resulting in a decline in its expression level or the corresponding protein function abnormal. It will make normal physiological function decline or loss, which will lead to the occurrence of a series of diseases, including the occurrence of malignant tumors^2^. In our previous study, we demonstrated that RNF180 protein expression in tumor tissues is significantly associated with the overall survival (OS) of 134 patients with GC after curative gastrectomy^3^. Studies have shown that high levels of vascular endothelial growth factor-C and D (VEGF-C and D) expression are closely related to the density of lymphatic vessels and lymph node metastasis in GC and significantly associated with the prognosis of these patients^4,5^. In addition, we found an obvious negative correlation between RNF180 protein expression in tumor tissues and lymph node metastasis stage in patients with GC^3^.

STAT3, an important oncogene is widely reported in many malignant tumors, and its activation involved in the development and progression of GC^6–8^. We previously demonstrated that the expression level of STAT3 in the GC tissues was significantly correlated with lymph node metastasis rate^9,10^. Moreover, we found that STAT3 knockdown could significantly reduce the protein expression of VEGF-C and reduce the expression of VEGF-D to some extent^11^. RNF180 and STAT3 have considerable roles in GC, but their specific mechanisms remain unclear. Considering that RNF180 and STAT3 are associated with lymph node metastasis in GC and could regulate the expression of VEGF-C/D, we suppose a certain relationship between these proteins. In this study, we utilized human tissues, ubiquitination label-free quantitative proteomic analysis, and in vivo and in vitro assays to investigate the possible role of RNF180 in regulating STAT3 in GC. We also conducted ubiquitination label-free quantitative proteomic analysis to identify the oncogene RhoC. The abnormally high expression of RhoC has been associated with the development of malignant tumors, such as head and neck cancers, ovarian cancer, and GC, and could promote the proliferation, invasion, and migration of cancer cells^12–14^. The results demonstrated that RNF180 has a critical role in mediating the activity of STAT3 by regulating RhoC expression via the proteasomal pathway in GC cells.

## Materials and methods

### Tissue sample collection and follow-up

We collected tumor and adjacent non-tumor tissues from 113 patients with GC who underwent curative gastrectomy between January 2004 and September 2007 at Tianjin Medical University Cancer Hospital (Tianjin, China), Xijing Hospital of Air Force Medical University (Xi’an, China), and Renji Hospital of Shanghai Jiao Tong University School of Medicine (Shanghai, China). After curative surgery, all patients received standard followed up according to the guideline. The median follow-up for the entire cohort was 36 months (range: 2–77 months). The follow-up of all patients included in this study was completed in December 2010. Patient consent was obtained for the use of the tissue samples. The study protocol and permission for the use of the clinical data were given by the Institutional Research Ethics Committee of Tianjin Medical University Cancer Institute and Hospital (Tianjin, China).

### Cell lines

Gastric cancer cell lines HGC-27 and SGC-7901 were purchased from Cancer Research Institute of Beijing, Beijing University, China. All cell lines were performed cell line authentication before receipt, and all of the cell lines were passaged for no >6 months. Cells were cultured in RPMI 1640 medium (Gibco, Carlsbad, CA, USA) supplemented with 10% fetal bovine serum (FBS) and maintained at 37 °C in a 5% CO_2_ atmosphere. HEK293T cell line was obtained from American Type Culture Collection (ATCC) and at 37 C in 5% CO_2_ in high glucose Dulbecco’s Modified Eagle’s medium (DMEM) supplemented with 10% FBS.

### Immunohistochemistry

The expression of RNF180 and RhoC was observed in 113 pairs of tumor and adjacent non-tumor tissues by Immunohistochemistry (IHC). Rabbit anti-RNF180 antibody (1:150) (GTX119301, GeneTex) and mouse anti-RhoC (1:50) (sc-393090, Santa Cruz Biotechnology) were used as primary antibodies. Positive cell rates and coloring intensity were scored separately. Then, the IHC staining score was obtained by using the *H*-score system. The cytoplasmic expression of RNF180 was assessed by assigning scores to the average intensity of positive tumor cells^15^.

### Western Blot

Western blot was performed following standard methods. The primary antibodies used for Western blot are as follows: rabbit anti-RNF180 antibody (1:1000) (GTX119301, GeneTex), mouse anti-RhoC (1:50) (sc-393090, Santa Cruz Biotechnology), rabbit anti-pSTAT3 antibody (1:2000) (9145, Cell Signaling Technology), rabbit anti-STAT3 antibody (1:1000) (12640, Cell Signaling Technology), rabbit anti-flag antibody (1:1000) (2272S, Cell Signaling Technology), rabbit anti-HA antibody (1:1000) (3724T, Cell Signaling Technology), rabbit anti-myc antibody (1:1000) (14793S, Cell Signaling Technology), and rabbit anti-β-actin antibody (1:1000) (GTX109639, GeneTex).

### Vector construction and transfection

The expression level of RNF180 was upregulated by using plasmid (pCMV6-AC-GFP-RNF180), and RhoC was knocked down by using short hairpin RNA (shRNA). An empty vector was transfected into the cells at the same time. Lipofectamine™ 3000 was used to transfect the shRNAs into the GC cells. Transfection efficiency was measured by using Western blot.

### Cell viability assay

Cell viability was assessed by performing cell viability assays with a cell counting kit 8 (CCK8). Cells were plated in a 96-well culture plate with 1 × 10^3^ cells per well, and 10 μL of CCK8 reagent was added to each well once a day. The optical density of the solution at 450 nm was measured after 3 h.

### Colony formation assay

Colony formation assay was used to detect the proliferation ability of GC cells. We seeded 1000 cells into each well of a 6-well plate and incubated at 37 °C for 12–14 days. The medium was changed at regular intervals until a macroscopic clone formed.

### Wound healing assay and transwell tumor cell invasion assay

A wound healing assay was performed on transfected GC cells as described in our previous study^16^. Matrigel™ (Sigma) invasion experiments were performed on transfected GC cells as described by Jingyu et al.^16^.

### Animal experiment

To explore the effect of RhoC on GC cell proliferation in vivo, we purchased seven female 4-week-old BALB/c mice (SPF [Beijing] Biotechnology Co., Ltd.). All experimental procedures were in accordance with the protocols approved by the Institutional Animal Care and Research Advisory Committee of Tianjin Medical University. All mice were bred in the animal facility of Tianjin Medical University Cancer Hospital. A total of 10^6^ HGC-7901-shControl and HGC-7901-shRhoC cells were subcutaneously xenografted into the mice. The length (*l*) and width (*w*) of the tumors were measured after 3 weeks, and the tumor volume was calculated as *v* = 1/2 × *l* × *w*^2^.

### Ubiquitination label-free quantitative proteomic analysis

HGC-27 cells were transfected with the RNF180 expression and empty vectors. Total proteins from each sample were extracted via SDT lysis and separated with 12.5% SDS-PAGE gel. Protein bands were visualized by Coomassie Blue R-250 staining. Peptides were collected with trypsin, desalted, concentrated, and reconstituted. The peptide content was estimated by determining the UV spectral density at 280 nm. Liquid chromatography–tandem mass spectrometry was performed at Shanghai Applied Protein Technology (Shanghai, China) using a Q Exactive mass spectrometer (ThermoScientific).

### Co-immunoprecipitation assay

Total proteins were extracted 72 h after HEK293T cells were co-transfected with the indicated plasmids. The protein lysates were incubated with anti-FLAG or anti-MYC agarose beads at 4 °C overnight, centrifuged at 3000 × *g* for 5 min, and then washed with lysis buffer thrice. Finally, the precipitates were collected for subsequent Western blot analysis. The plasmid of HA-ubiquitin was co-transfected into HEK293T cells according to the above experimental methods, and precipitates were collected for subsequent Western blot analysis.

### Protein stability and degradation experiment

Cycloheximide (CHX), a protein synthesis inhibitor, could act on the large subunit of the eukaryotic ribosome, inhibit transpeptidase, block peptide chain extension, and further inhibit protein synthesis. Approximately 30 h after transfection, CHX (HY12320; MedChemExpress) was added to the GC cell culture medium at a concentration of 100 μg/mL, and the total protein was extracted for the western blot analysis. MG132, a proteasome inhibitor, could inhibit protein degradation in a proteasome-dependent manner. Approximately 48 h after transfection, 5 μM MG132 (HY12359; MedChemExpress) was added to the GC cell culture medium, and the same amount of dimethyl sulfoxide (DMSO) was added to the control group. Then, 11 h after this treatment, the total protein was extracted for Western blot analysis.

### Statistical analysis

We analyzed the results by using *t*-test to compare statistical differences between two groups or a one-way analysis of variance to compare differences among the three groups. Statistical analyses were performed by using IBM SPSS Statistics (version 24.0; Armonk, NY, USA) and GraphPad Prism Version 8.0 software. Overall survival (OS) was using the Kaplan–Meier method and a log-rank test performed to determine significance. The multivariate analysis of OS was performed by the Cox proportional hazard model with forwarding step procedures. *P* value < 0.05 was considered statistically significant.

## Results

### Patient outcomes related to RNF180 expression

**Fig. 1:**
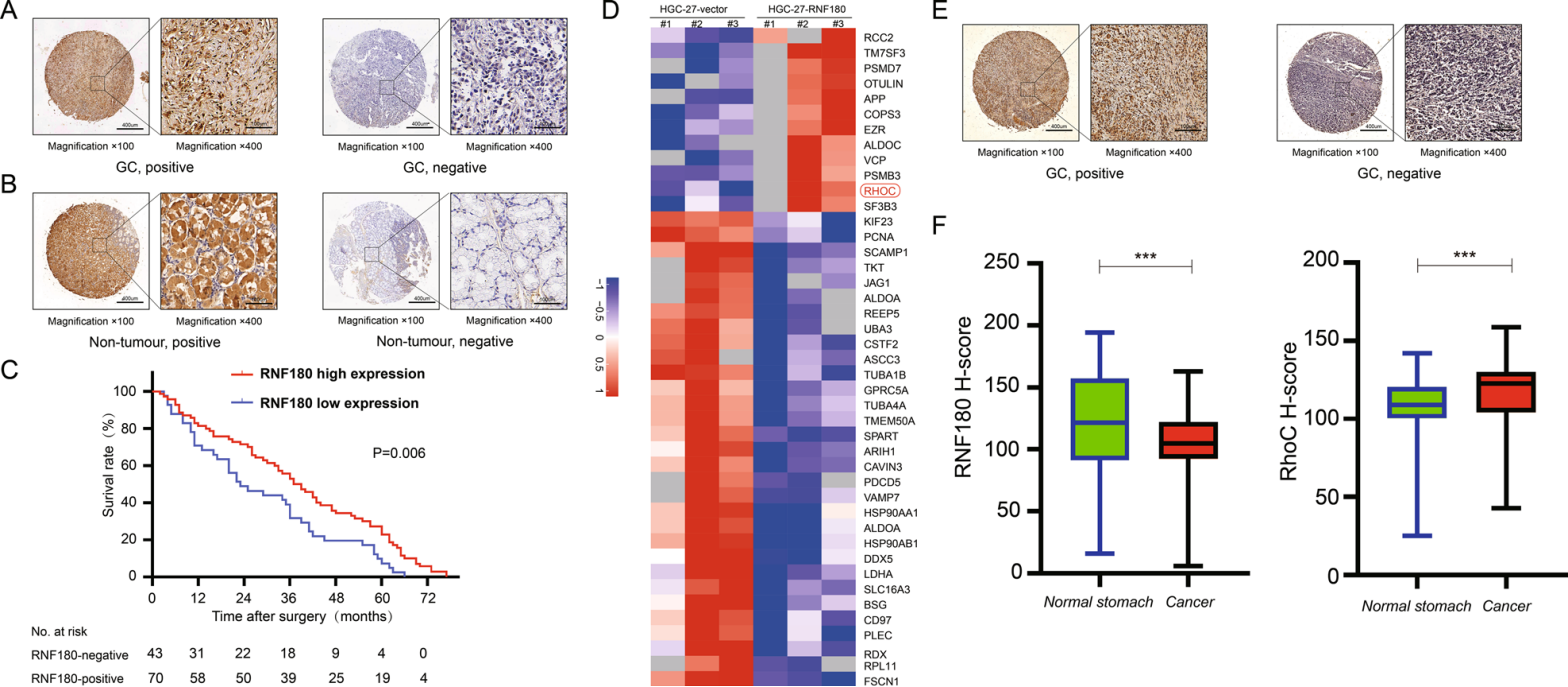
Immunohistochemical (IHC) staining images of RNF180 and RhoC using human gastric cancer tissue microarrays that contained 113 cancer samples with their matching adjacent histologically normal stomach, original magnification is at ×100 and insets are at ×400. **A**, **B** Examples of RNF180 immunohistochemical staining Showing. **C** Kaplan–Meier survival curves according to the expression of RNF180 in GC tissues. *P* = 0.006 (log-rank test). **D** Ubiquitination label-free quantitative proteomic analysis revealed the ubiquitination level proteins upregulating RNF180 compared with the control group. **E** Examples of RhoC immunohistochemical staining showing. **F** Summary graphs of the IHC staining of RNF180 and RhoC results using box-and-whisker blots to depict the smallest value, lower quartile, median, upper quartile, and largest value.

We measured the expression of RNF180 in 113 pairs of tumor and adjacent non-tumor tissues by IHC (Fig. 1A–B). The results of univariate and multivariate survival analyses of 113 patients with GC are summarized in Table 1. The median overall survival (OS) of all patients with GC was 36 months, and 25 patients were alive at the end of follow-up. Kaplan–Meier analysis showed that patients with negative (no/weak) RNF180 protein expression had poorer survival than those with positive (moderate/high) RNF180 protein expression (Fig. 1C). RNF180 protein expression in GC tissues was identified as an independent predictor of outcome (hazard ratio [HR] 0.628; *P* = 0.006), pN stage (HR 1.258; *P* = 0.005) and tumor location (HR 1.275; *P* = 0.015). RNF180 negativity indicated a tendency of poor prognosis in patients with GC.

**Table 1:** Table characteristics of the study cohort.

Characteristics	Case	5-YSR (%)	*P*-value^†^	Hazard ratio (95 % CI)	*P*-value^‡^
*Gender*			0.269		
Male	78	36.60			
Female	35	31.97			
*Age at surgery (years)*			0.178		
<65	71	37.68			
≥65	42	30.93			
*Tumor location*			0.015	1.275 (1.014–1.603)	0.038
Upper third	17	39.06			
Middle third	15	40.73			
Lower third	51	38.10			
>2/3 stomach	30	26.217			
*Lauren classification*			0.683		
Intestinal	29	33.83			
Diffuse	84	35.63			
*Tumour size (cm)*			0.055		
<4	19	50.21			
≥4	94	32.13			
*Type of gastrectomy*			0.299		
Total	42	30.60			
Proximal subtotal	14	36.21			
Distal subtotal	57	38.28			
*Soft tissue invasion*			0.093		
No	77	32.14			
Yes	36	41.64			
*pT stage*			0.964		
pT2	7	34.29			
pT3	9	35.78			
pT4a	90	34.99			
pT4b	7	37.57			
*pN stage*			0.005	1.258 (1.092–1.450)	0.002
pN0	23	51.52			
pN1	14	37.93			
pN2	25	32.40			
pN3a	26	28.12			
pN3b	25	28.68			
RNF180 expression*			0.006	0.628 (0.405–0.974)	0.038
Low	43	29.72			
High	70	38.51			

Values in parentheses are 95 percent confidence intervals.

*Determined by immunohistochemical staining.

^†^Log-rank test.

^‡^Cox proportional hazards model.

### Upregulation of RNF180 increased the ubiquitination level of RhoC

To determine substrates that may be regulated by RNF180, we measured the ubiquitination level of proteins extracted from HGC-27-vector and HGC-27-RNF180 cells. Ubiquitination label-free quantitative proteomic analysis revealed that the ubiquitination level of 12 proteins is significantly increased compared with the control group by upregulating RNF180 (fold change ≥ 1.5, *P* < 0.05) (Fig. 1D). Among these 12 proteins, we chose RhoC for further analysis because previous reports indicated that the expression of RhoC is associated with metastasis of gastric cancer^17^.

### RNF180 and RhoC expression patterns in human tissue microarrays

We measured the protein expression levels of RNF180 and RhoC in 113 pairs of tumor and adjacent non-tumor tissues by IHC (Fig. 1E). RNF180 protein expression levels in GC were significantly lower than those in normal gastric tissues (*P* = 0.0002). By contrast, GC tissues showed higher expression levels of RhoC compared with normal gastric tissues (*P* = 0.0011). The IHC analysis results of RNF180 and RhoC are summarized in Fig. 1F, respectively.

### RNF180 promotes RhoC degradation

**Fig. 2:**
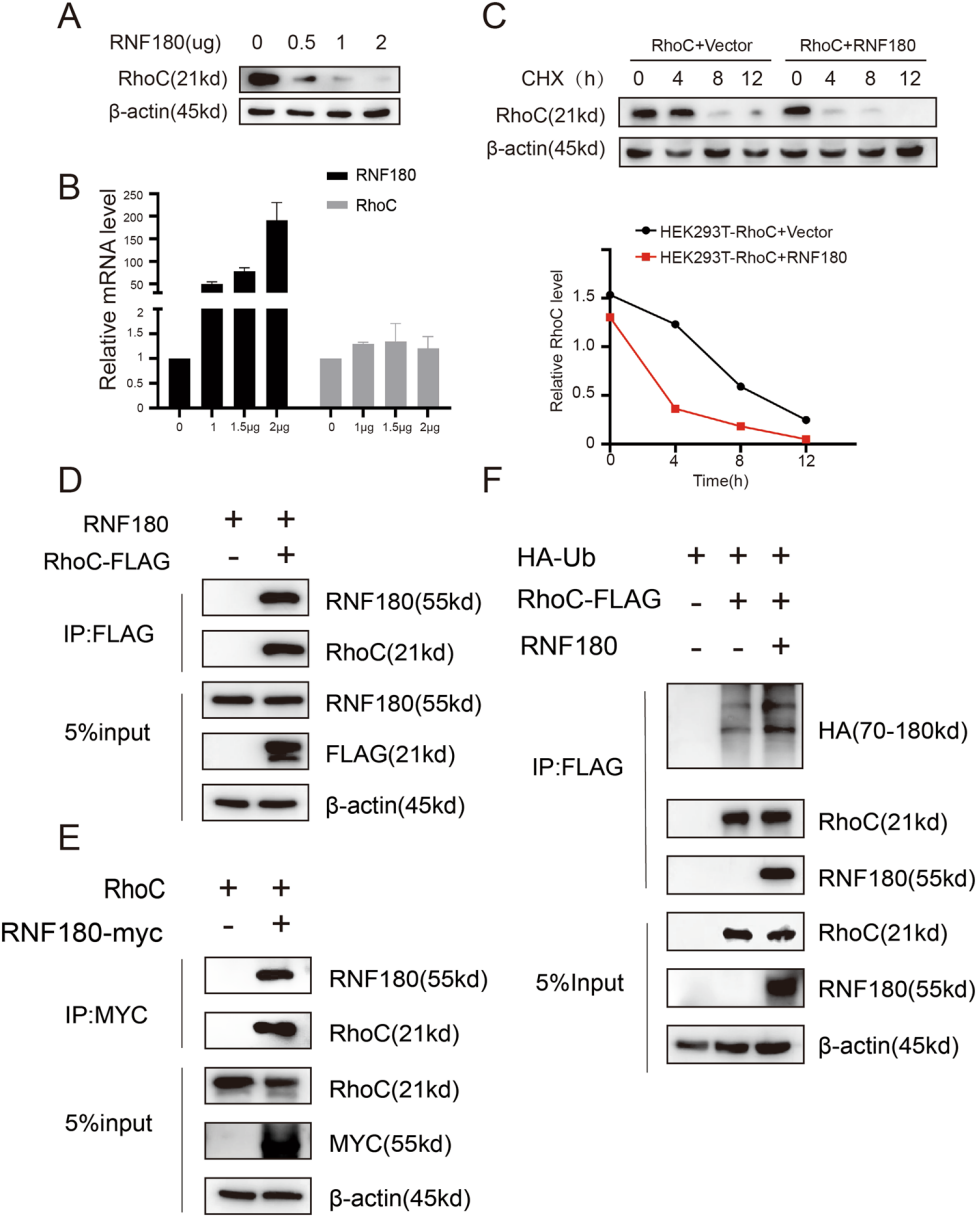
RNF180 promotes the degradation of RhoC protein by increasing its ubiquitination level and interacts with RhoC. **A** Increasing concentrations of RNF180 plasmid were transfected into HEK293T cells for 36 h and the protein level RhoC were analyzed by immunoblotting. **B** The mRNA level of RNF180 and RhoC were analyzed by RT-qPCR assay. **C** HEK293T cells were co-transfected by RhoC plasmid together with either RNF180 plasmid or control vector. After 36 h, cells were treated with 100 μg/ml CHX at the indicated time point. The cell lysates were subjected to immunoblotting and RhoC expression was quantified by ImageJ software. **D** The RNF180 plasmid was co-transfected transiently with RhoC-FLAG plasmid or control vector into HEK293T cells and 36 h later, cells were incubated with 5 μM MG132 for 6 h. Cell lysates were immunoprecipitated to pull down RhoC by the FLAG M2 affinity gel and subjected to immunoblotting. 5% of cell lysates were used to examine the total expression of RhoC and RNF180. **E** RNF180-FLAG plasmid or control vector was co-transfected transiently with the RhoC plasmid into HEK293T cells and the co-immunoprecipitation assay was performed as described in **D**. **F** HA-Ub and RhoC-FLAG plasmids were co-transfected with RNF180 in HEK293T cells. After 36 h, cells were treated with 5 μM MG132 for 6 h. RhoC-FLAG protein was immunoprecipitated and analyzed by immunoblotting. The poly-ubiquitination level of RhoC was detected by the anti-HA antibody.

RNF180 has been demonstrated to be a ubiquitin ligase for several substrates. To support our hypothesis that RNF180 could also function as a ubiquitinating ligase to promote RhoC degradation, we transfected increasing concentrations of RNF180 into HEK293T cells. The protein level of RhoC was gradually reduced by RNF180 in a dose-dependent manner (Fig. 2A). However, RhoC mRNA levels did not show significant differences (Fig. 2B), thereby indicating that RNF180 regulates RhoC expression at the posttranslational level. CHX pulse-chase assay was performed to examine the function of RNF180 in RhoC. We observed that RNF180 overexpression could remarkably decrease RhoC protein levels and reduce its half-life (Fig. 2C). To confirm whether RhoC is a direct substrate of RNF180, we performed a co-immunoprecipitation assay. As shown in Fig. 2D, RhoC is a direct substrate of RNF180. A reciprocal co-immunoprecipitation experiment was then carried out to further define their interaction (Fig. 2E), and an in vivo ubiquitination assay was performed to confirm whether the ubiquitination level of RhoC is greatly increased by RNF180 (Fig. 2F). Taken together, our findings indicate that RNF180 is a ubiquitin ligase targeting RhoC for ubiquitination and degradation.

### RNF180 regulates RhoC stability in GC

**Fig. 3:**
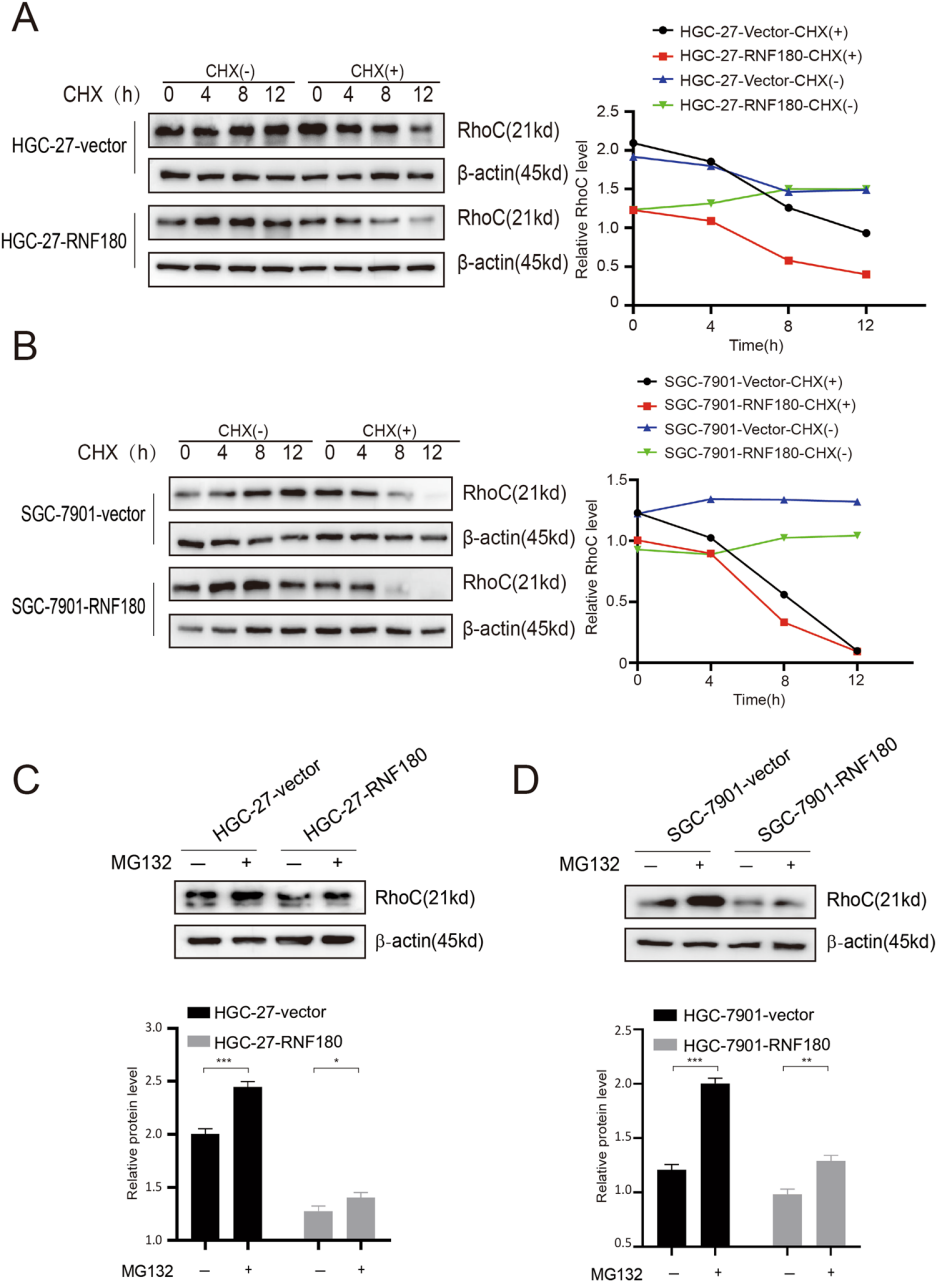
RNF180 promotes the degradation of RhoC protein by increasing its ubiquitination level in gastric cancer cells. **A**, **B** GC cells were transfected by either RNF180 plasmid or control vector. After 36 h, cells were treated with 100 μg/ml CHX at the indicated time point. The cell lysates were subjected to immunoblotting and RhoC expression was quantified by ImageJ software. **C**, **D** Ubiquitination assay was performed with 5 μM MG132 for 11 h in order to further explore the ubiquitination degradation of RhoC and pSTAT3 by RNF180 in GC cells.

We explored the influence of RNF180 on the degradation of RhoC protein by utilizing CHX, which could inhibit protein synthesis. The results showed that the protein band gradually weakens with the passage of time after CHX treatment and that this phenomenon is more pronounced in the RNF180 group than in the vector group in HGC-27 and SGC-7901 cells (Fig. 3A, B). Ubiquitination assay was performed with MG132 to explore the ubiquitination and degradation of RhoC by RNF180. As shown in Fig. 3C, D, the protein expression of RhoC in cells treated with MG132 was higher than that in cells treated with DMSO, thereby indicating that RNF180 could promote RhoC degradation through the ubiquitin–proteasome system.

### RhoC enhances the proliferation, migration, and invasion of GC cells and tumor growth in mice

**Fig. 4:**
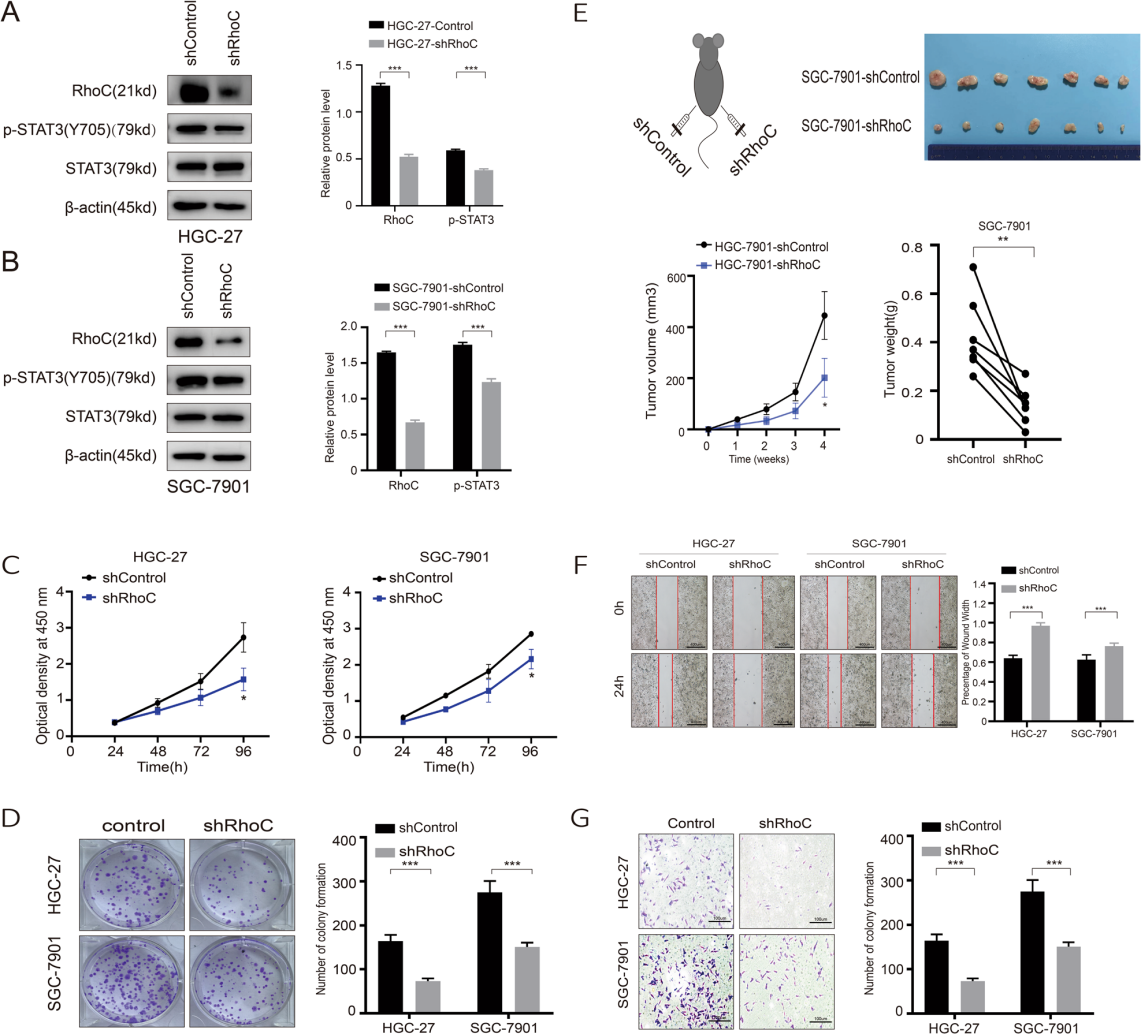
RhoC activity is required for tumor cell proliferation and migration. HGC-27 and SGC-7901 cells were treated with either a control shRNA or an shRNA targeting RhoC. Stably transduced cells were used for the analysis of protein expression by western blotting and cell function. **A**, **B** The protein levels in these established cell lines were verified by western blot. **C** CCK8. **D** Colony formation assay. **E** Xenograft tumor formation of SGC-7901 in nude mice. **F** Cell-based scratch assay, **G** transwell tumor cell invasion assay.

To investigate the biological function of RhoC in GC cells, we used shRNA to transfect HGC-27 and SGC-7901 cells and downregulate RhoC. The empty vector was also transfected as a control. As shown in Fig. 4A, B, protein expression levels were significantly decreased after plasmid transfection. The cell growth curve of CCK8 assay results showed that downregulated RhoC suppressed cell proliferation and viability (Fig. 4C). Similarly, as shown in Fig. 4D, fewer colonies were formed in the downregulated RhoC group than in the control group. Thus, cell viability was significantly weaker in the downregulated RhoC group than in the vector group. The size and weight of the subcutaneous tumor transfected with shRNA RhoC was significantly decreased than that in tumor transfected with an empty vector. RhoC appeared to remarkably enhance tumor growth in vivo (Fig. 4E). Wound healing and transwell assays were conducted to investigate the effect of RhoC on the migration and invasion ability of HGC-27 and SGC-7901 cells (Fig. 4F, G). Wound healing and transwell assays result showed that the migration distances of the downregulated RhoC group were significantly reduced compared with that of the control group. Thus, the results revealed that the proliferation and migration ability of cells in the downregulated RhoC group was lower than that in the vector group.

### RNF180 inhibits constitutively active STAT3 in GC

**Fig. 5:**
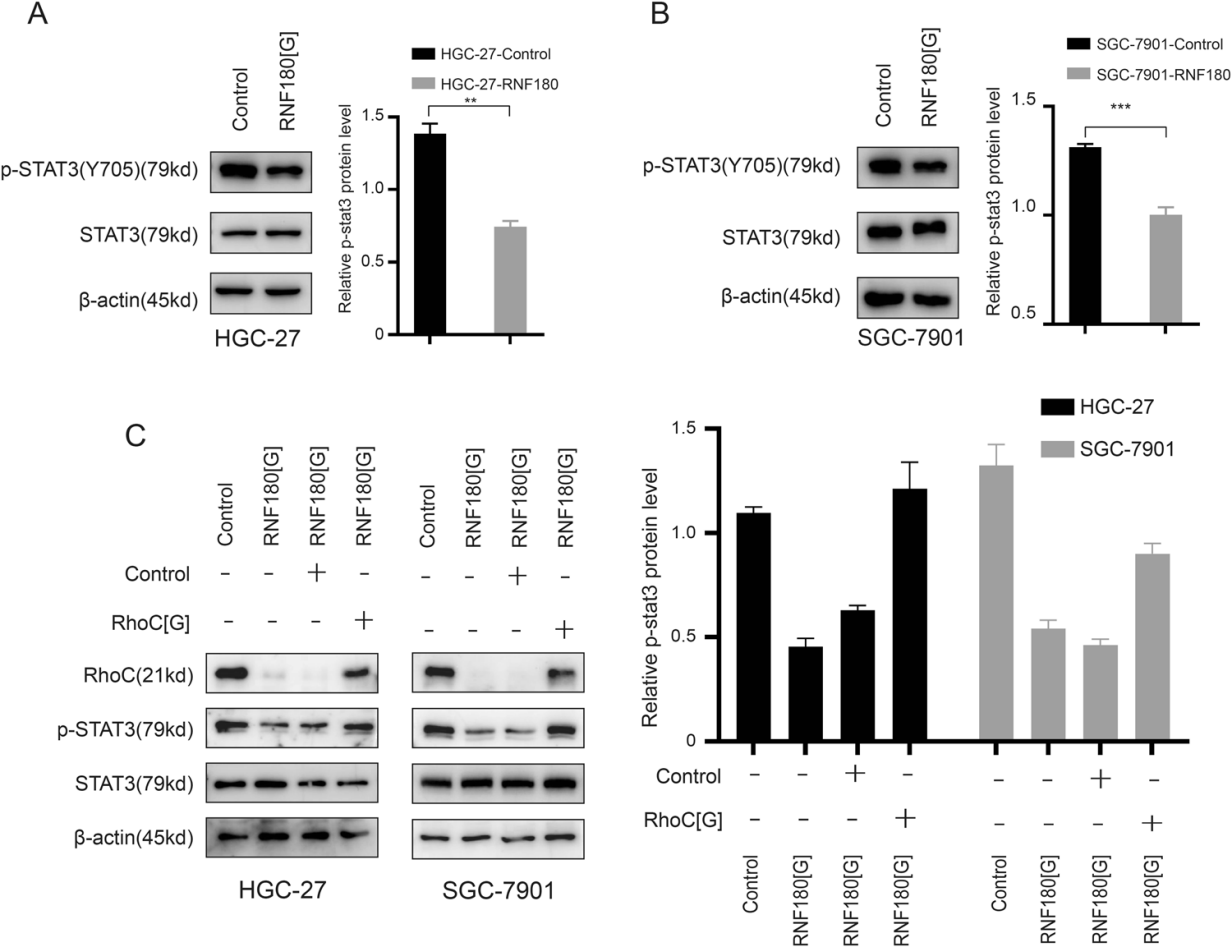
RhoC antagonizes RNF180-mediated suppression of p-STAT3 activation in gastric cancer cells. **A**, **B** HGC-27 and SGC-7901 cells RNF180 compared with control GC cells were established by transiently transfection, respectively. The protein levels in these established cell lines were verified by western blot at 48h after transfection. **C** The rescue of RhoC and pSTAT3 that resulted from overexpression of RNF180 by overexpression of RhoC.

**Fig. 6:**
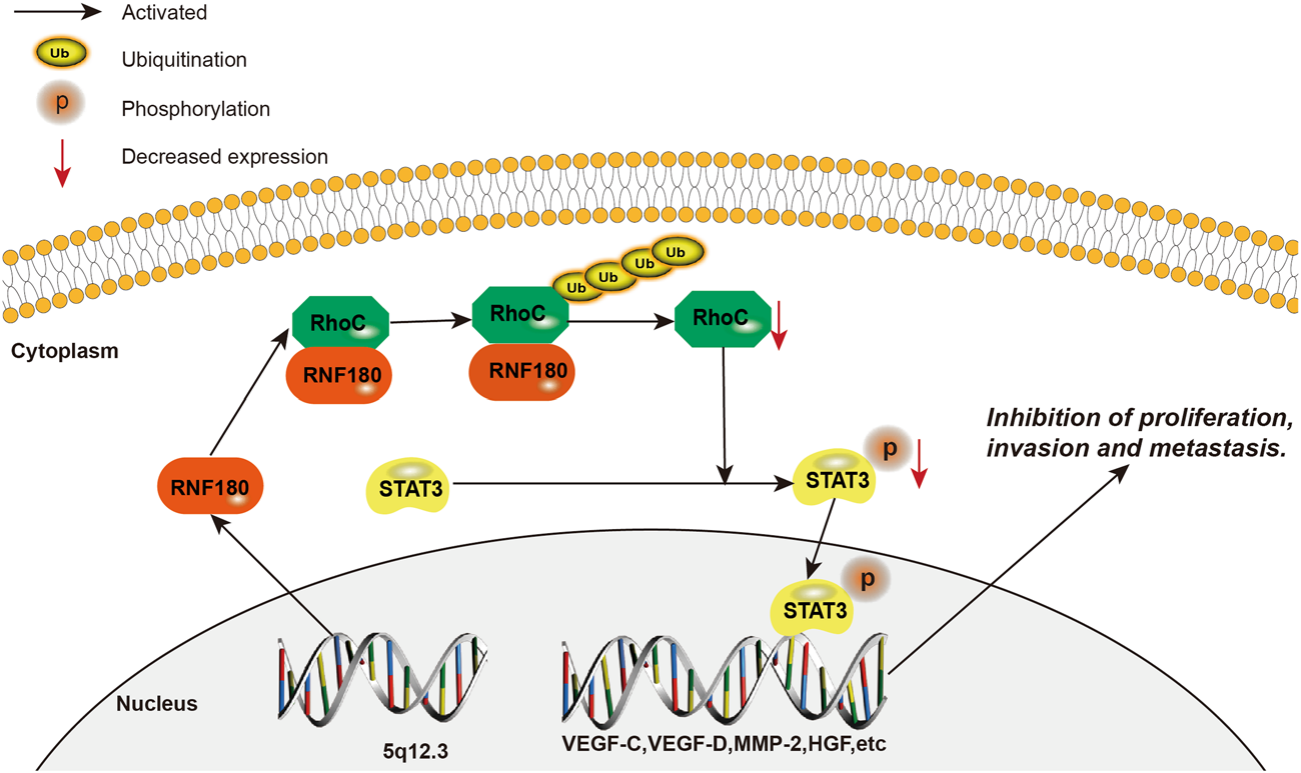
Model depicting the role of RNF180 in regulating the expression of protein level of pSTAT3(Y705). Overexpression of RNF180 promotes the degradation of RhoC protein by increasing its ubiquitination level. RhoC could also reduce the protein level of pSTAT3(Y705). Moreover, a low level of pSTAT3 could efficiently inhibit the proliferation and invasion of gastric cancer.

Persistent activation of STAT3 contributes to uncontrolled cell proliferation, angiogenesis, apoptotic resistance, and prosurvival effects in cancer cells^18^. Western blot analysis of HGC-27 and SGC-7901 cells showed that the protein expression level of pSTAT3 (Y705) is obviously downregulated by RNF180 (Fig. 5A, B). In addition, knockdown of RhoC could reduce the activation level of STAT3 but not that of STAT3 (Fig. 4A, B). Inhibition of pSTAT3 resulting from RNF180 overexpression could be rescued by RhoC overexpression (Fig. 5E), likely because STAT3 is known as a RhoC downstream molecule with a critical role in regulating the interferon response pathway^12^. Based on the above results, we believe that RNF180 could inhibit STAT3 activation by degrading RhoC. We observed altered mRNA expression levels in genes downstream of the STAT3 signaling pathway in GC cells featuring STAT3 knockdown, including substantial decreases in matrix metalloproteinase (MMP)-2, MMP-14, VEGF-C, VEGF-D, and hepatocyte growth factor (HGF) levels compared with those in control GC cells (Supplementary Fig 1). A schematic summarizing the effects of RNF180 on the inhibition of GC proliferation is shown in Fig. 6.

In summary, our findings suggest that RNF180 could reduce tumor metastasis in GC by inhibiting STAT3 activation via RhoC protein degradation through the ubiquitin–proteasome system.

## Discussion

In this study, we utilized human tissues, ubiquitination label-free quantitative proteomic analysis, and in vivo and in vitro assay to examine the role of RNF180 in suppressing STAT3 activity by degrading RhoC protein through the ubiquitin–proteasome system.

The ubiquitin–proteasome system is one of the major systems involved in proteolysis in eukaryotic cells^19^. E3 ubiquitin ligase is considered the most important component of this system because it can recognize substrates specifically^20^. Our own studies on RNF180 revealed that the newly discovered E3 ubiquitin ligase could inhibit the lymph node metastasis of GC^3^. In the present study, our findings demonstrated that RNF180 could bind with RhoC and increase its ubiquitination to promote protein degradation. We also found that the protein level of RhoC in GC cells decreases gradually after the addition of CHX and that this phenomenon is more pronounced in the RNF180 upregulation group. The addition of the proteasome inhibitor MG132 inhibited the degradation of RhoC, which means RNF180 could modulate the proteolysis of RhoC through the ubiquitin–proteasome pathway.

RhoC is a guanosine triphosphatase that can modulate the proliferation, differentiation, and apoptosis of normal cells^21–23^. Previous studies have shown that the Rho-family of small GTPases has an essential role in transmitting the VEGF signals downstream to angiogenesis^24^. Upon VEGF stimulation, Src interacts with FAK and phosphorylated at Tyr-416. Then, the Src/FAK complex further activates Rho-GTPases^25^. The super-family of Rho-GTPases consists of various Rho specific insertion domains containing subfamilies such as Rho, Rac, Cdc-42, etc. RhoC expression and/or activity frequently is increased in various cancers^26^. Moreover, increased expression of RhoC may be involved in the metastasis of gastric cancer, and RhoC appears to be a good genetic marker of a metastatic potential^21^. Islam et al.^12^ found that RhoC could regulate cancer stem cells (CSCs) by overexpressing the phosphorylation of STAT3. To further understand how RhoC regulates the expression of the GC cell transcription factors, we analyzed expression levels of the STAT3. Since the role of RhoC in STAT3 phosphorylation has not been established before in GC, we analyzed the phosphorylation of STAT3 tyr705 in the RhoC knockdown cell lines and the control and showed that there were a decrease in pSTAT3 tyr705 in the RhoC knockdown GC cell lines (Fig. 4A, B). Based on these results, we could conclude that RhoC promoted the proliferation and migration of GC cells through regulating the phosphorylation of STAT3. Apparently, knockdown of RhoC suppressed tumor proliferation and invasion (Fig. 4C–G). Taken together, in this study, RNF180 is characterized as a tumor suppressor to inhibit tumor metastasis by reducing RhoC in gastric cancer.

Metastasis is closely associated with poor survival outcomes. As a well-known transcription factor, STAT3 is implicated in the proliferation, migration, and invasion of various tumor cells depending on its active form, pSTAT3^27^. Activation of STAT3 transcription activity induces the expression of a wide range of target genes promoting key pro-oncogenic cellular functions, such as inflammation, proliferation, survival, invasion, and angiogenesis^28^. STAT3 is activated by phosphorylation of the Y705 position, followed by nuclear translocation of the phosphorylated proteins with subsequent activation of the transcription of specific downstream genes and metastasis of cancers^29^. Our results demonstrated decreased mRNA expression levels in GC cells featuring STAT3 knockdown and downregulation of several STAT3 target genes, such as MMP-2, MMP-14, VEGF-C, VEGF-D, and HGF. These genes have an important role in regulating cellular functions in cancer development and progression.

Based on our findings, we constructed the signaling pathway by which RNF180 regulates the proliferation and invasion of gastric cancer (Fig. 6). RNF180 binds to the RhoC and degrade of RhoC protein by increasing its ubiquitination level, which can phosphorylate STAT3 and increase the protein level of pSTAT3(Y705). Phosphorylated STAT3 at tyr705 is able to dimerize and then diffuse into the nucleus where it binds to the promoter region of nanog to switch on its expression. As a result, a low level of pSTAT3 could efficiently inhibit the proliferation and invasion of gastric cancer. In this way, increased RNF180 expression in GC results in the inhibition of the core cell transcription factors that are needed for their proliferation and invasion. This in turn significantly decreases the tumors’ ability to grow and metastasize to other body regions. The implications of these findings provide a fertile area of research in GC. Additional studies are needed to understand how RNF180 regulates the RhoC expression and what additional signaling molecules are involved.

In summary, our study reveals that RhoC is a substrate of RNF180 and that ubiquitination and degradation of RhoC by RNF180 could inhibit proliferation and invasion of gastric cancer. Thus, RNF180 may be a potential candidate for GC treatment, illustrating that RNF180 has an important role in gastric cancer and its propagation by modulating the phosphorylation state of STAT3. Considering the low expression of RNF180 in gastric cancer, efficiently inhibit RhoC expression might repress tumorigenesis and tumor metastasis and then improve the survival outcomes of patients, which still need further investigation. It seems to be a promising cure for the metastatic tumors to develop small molecule inhibitors for RNF180 to target RNF180-decreased RhoC. With additional investigations and ongoing development of molecular therapy for gastric cancer, this may prove to be an important therapeutic target in the GC patient population.

## Supplementary information

Supplementary Table, figure legend and method

Supplementary figure
