# Supplementary material for: RNF180 mediates STAT3 activity by regulating the expression of RhoC via the proteasomal pathway in gastric cancer cells
Source: Cell Death Dis. 2020 Oct 20;11(10):881. doi: 10.1038/s41419-020-03096-3 (PMC7575565; doi:10.1038/s41419-020-03096-3)
Supplement: Supplementary file 1 — Supplementary Table, figure legend and method [file 41419_2020_3096_MOESM1_ESM.doc]

**Supplementary Table: Primers utilized for target genes detection in cell lines**

| **Genes** | **Primer sequences** | **Length(bp)** |
| --- | --- | --- |
| RNF180 | 5’-GGCAGGCAGACTAATGAGACCATC/AGCCACCACCTGTCAGCAGAG-3’ | 95 |
| RhoC | 5’-ACCTGCCTCCTCATCGTCTTCAG/GCTTGCCGTCCACCTCAATGTC-3’ | 100 |
| STAT3 | 5’-CACCAAGCGAGGACTGAGCATC/AGCCAGACCCAGAAGGAGAAGC-3’ | 150 |
| VEGF-C | 5’-CCACAGTGTCAGGCAGCGAAC/CTGTTGAGTCATCTCCAGCATCCG-3’ | 121 |
| VEGF-D | 5’-GGGCTGCTTCTAGTTTGGAGGAAC/TAGTGGACCGATGGGATGCTGAG-3’ | 129 |
| MMP-14 | 5’-GCCTGCCTGCGTCCATCAAC/GCCTCATCAAACACCCAATGCTTG-3’ | 91 |
| MMP-2 | 5’-TGCGGCACCACTGAGGACTAC/GCACCTTCTGAGTTCCCACCAAC-3’ | 92 |
| HGF | 5’-TGTGCTGGGGCTGAAAAGATTGG/CCACGACCAGGAACAATGACACC-3’ | 116 |

**Supplementary Figure legend**

mRNA expression levels for genes downstream of STAT3 signaling in GC cells knockdown SATA3 compared with control GC cells.

**Supplementary methods**

**Quantitative Reverse Transcription-Polymerase Chain Reaction (qRT-PCR).**

Total RNA was isolated from cells by using TRIzol reagent (15596018, Thermo Fisher). Primescript™ RT Master Mix (RR036A; Takara) was used to synthesize the complementary DNA. 2 µg of RNA was added to the real time PCR, with the final primer concentration being 0.5 µM. The PCR was performed under the following condition: reverse transcription at 42 ◦C for 60 min, amplification for 30 cycles at 94 ◦C for 30 s, 58 ◦C for 50 s, and 72 ◦C for 50 s. The primer sequences used are given in Supplementary Table.
