# Supplementary figures and images for: RNF180 mediates STAT3 activity by regulating the expression of RhoC via the proteasomal pathway in gastric cancer cells
Source: Cell Death Dis. 2020 Oct 20;11(10):881. doi: 10.1038/s41419-020-03096-3 (PMC7575565; doi:10.1038/s41419-020-03096-3)

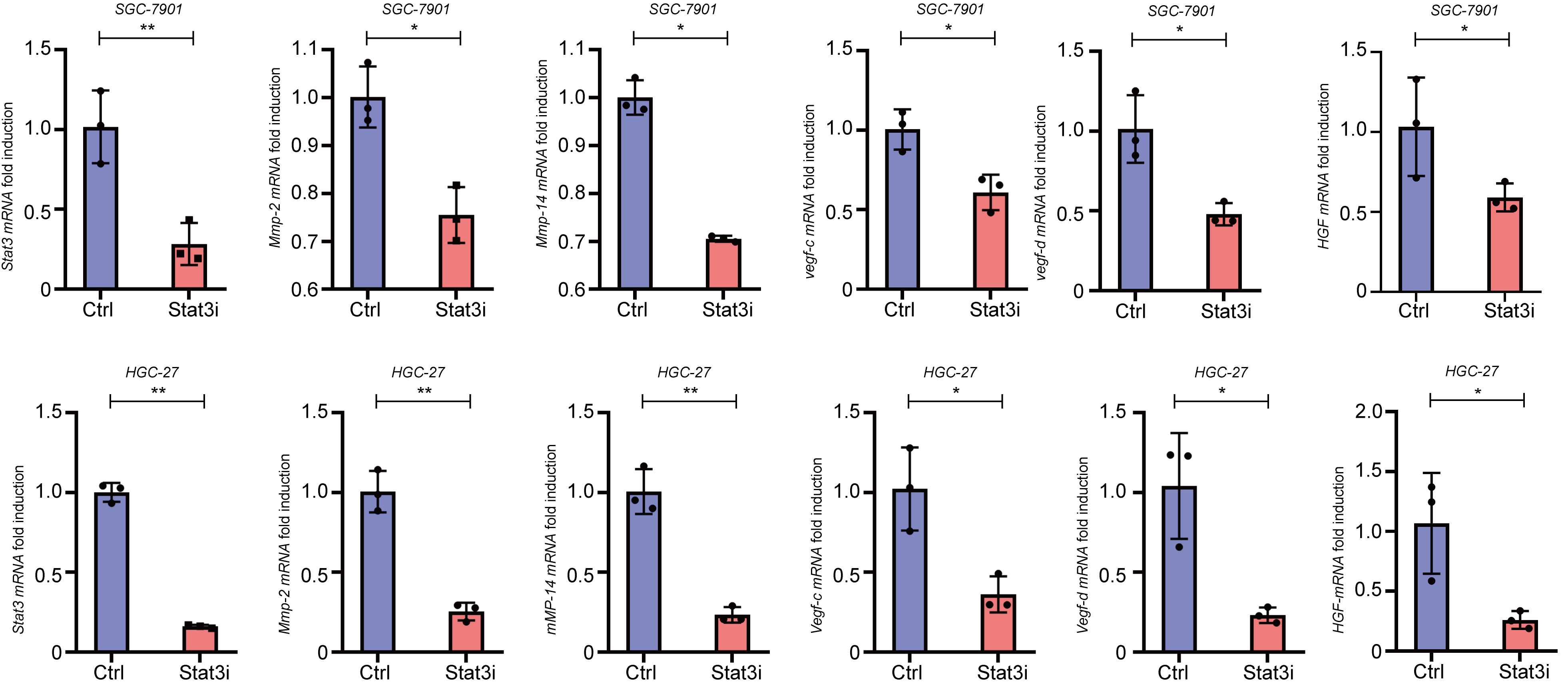

Supplement: Supplementary file 2 — Supplementary figure [file 41419_2020_3096_MOESM2_ESM.tif]
